# Supplementary material for: Are affective factors related to individual differences in facial expression recognition?
Source: R Soc Open Sci. 2020 Sep 9;7(9):190699. doi: 10.1098/rsos.190699 (PMC7540805; doi:10.1098/rsos.190699)
Supplement: Output and analysis code [file rsos190699supp1.pdf]

Data Processing
Analysis
Robustness Checks
Exploratory analyses of social anxiety

# Emotion Recognition RR

Code

Code

## Sample Size

Code

Sample size is set at $n = 160$, following the small telescopes approach, which sets replication sample size at 2.5 times the original sample size ($n = 63$). With $alpha = .05$, this design has 80% power for two-tailed tests to detect correlations as small as $|r| = 0.219$.

# Data Processing

## Simulate data

Descriptive data are taken from from Palermo et al (2018) with correlation structure from correlations reported in Palermo et al. (2018) or set to r = 0.5 for all depression/anxiety measures (as unreported in Palermo).

Code

## Load Data

Code

```
## Parsed with column specification:
## cols(
##   session_id = col_double(),
##   project_id = col_double(),
##   exp_id = col_double(),
##   user_id = col_double(),
##   user_sex = col_character(),
##   user_status = col_character(),
##   user_age = col_double(),
##   trial_name = col_character(),
##   trial_n = col_double(),
##   order = col_double(),
##   dv = col_character(),
##   rt = col_double(),
##   side = col_double(),
##   dt = col_datetime(format = "")
## )
```

Code

### Process questionnaire data

Code

### Filter data

Remove test runs and fix IDs

Code

```
## character(0)
```

Code

```
## character(0)
```

Code

```
## # A tibble: 0 x 2
## # … with 2 variables: ID_idcode <chr>, n <int>
```

### Calculate scores

Recode AQ and EQ

0 = definitely agree
1 = slightly agree
2 = slightly disagree
3 = definitely disagree

AQ: “Definitely agree” or “slightly agree” responses scored 1 point, on the following items: 1, 2, 4, 5, 6, 7, 9, 12, 13, 16, 18, 19, 20, 21, 22, 23, 26, 33, 35, 39, 41, 42, 43, 45, 46. “Definitely disagree” or “slightly disagree” responses scored 1 point, on the following items: 3, 8, 10, 11, 14, 15, 17, 24, 25, 27, 28, 29, 30, 31, 32, 34, 36, 37, 38, 40, 44, 47, 48, 49, 50.

Data Processing
Analysis
Robustness Checks
Exploratory analyses of social anxiety

EQ: “Definitely agree” responses scored 2 points and “slightly agree” responses scored 1 point on the following items: 1, 6, 19, 22, 25, 26, 35, 36, 37, 38, 41, 42, 43, 44, 52, 54, 55, 57, 58, 59, 60. “Definitely disagree” responses scored 2 points and “slightly disagree” responses scored 1 point on the following items: 4, 8, 10, 11, 12, 14, 15, 18, 21, 27, 28, 29, 32, 34, 39, 46, 48, 49, 50.

Code

Code

```
## Warning: Removed 17 rows containing non-finite values (stat_bin).
```

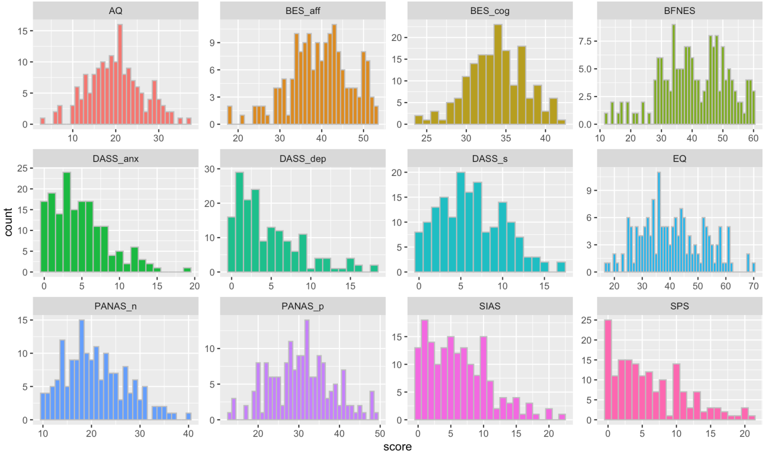


Code

```
## Warning: Removed 17 rows containing non-finite values (stat_bin).
```

### Process Experiment Data

Code

Code

```
## $expected
## [1] "026sad_fea01" "076ang_sid04" "123sur_fea11"
##
## $observed
## [1] "076ang_dis04"  "026sad_fea09"  "123sur_fear11"
```

Code

Code

```
## $expected
## [1] "023bm29anhl" "044bm11sahl" "074bf09afs"
##
## $observed
## [1] "044af22sahl" "074af07afs"  "023bm06anhl"
```

Code

### Calculate scores

Code

### Process MERT data

Code

Code

Code

| value | A | E | F | H | K | P | T | U | V | Z |
|---|---|---|---|---|---|---|---|---|---|---|
| Anxiety | 362 | 86 | 109 | 24 | 43 | 217 | 99 | 66 | 6 | 124 |
| Cold anger | 178 | 94 | 44 | 65 | 568 | 36 | 40 | 5 | 222 | 22 |
| Contempt | 62 | 132 | 154 | 18 | 378 | 11 | 61 | 13 | 244 | 9 |
| Despair | 95 | 42 | 39 | 11 | 43 | 162 | 274 | 90 | 16 | 441 |
| Disgust | 59 | 819 | 19 | 28 | 112 | 17 | 31 | 9 | 139 | 17 |
| Elated joy | 5 | 6 | 224 | 5 | 14 | 20 | 6 | 856 | 11 | 9 |

Data Processing
Analysis
Robustness Checks
Exploratory analyses of social anxiety

| value | A | E | F | H | K | P | T | U | V | Z |
|---|---|---|---|---|---|---|---|---|---|---|
| Happiness | 8 | 26 | 980 | 3 | 27 | 10 | 4 | 260 | 5 | 3 |
| Hot anger | 36 | 35 | 2 | 975 | 62 | 67 | 3 | 16 | 14 | 43 |
| Panic fear | 281 | 26 | 21 | 42 | 7 | 719 | 43 | 111 | 1 | 463 |
| Sadness | 90 | 78 | 88 | 5 | 90 | 85 | 951 | 86 | 14 | 212 |

### Recode MERT

"A" = "Anxiety", "K" = "Cold anger", "V" = "Contempt", "Z" = "Despair", "E" = "Disgust", "U" = "Elated joy", "F" = "Happiness", "H" = "Hot anger", "P" = "Panic fear", "T" = "Sadness"

Code

### Combine Data

Code

### Exclusions

Outliers (scores on a measure that are more than 3 standard deviations from the mean score for that measure) will be adjusted to a score one point higher than the closest non-outlier score (following Palermo et al, 2018). As a positive control, participants scoring lower than chance on either of the emotion recognition tasks will be excluded from all analyses.

Code

3 participants were removed who did not complete an emotion task and 9 participants who had missing questionnaire data.

Code

```
## # A tibble: 15 x 2
##    subscale  changed
##    <chr>       <int>
##  1 AQ              0
##  2 BES_aff         0
##  3 BES_cog         0
##  4 BFNES           0
##  5 DASS_anx        1
##  6 DASS_dep        1
##  7 DASS_s          0
##  8 emo_label       3
##  9 emo_match       2
## 10 EQ              0
## 11 MERT            2
## 12 PANAS_n         0
## 13 PANAS_p         0
## 14 SIAS            1
## 15 SPS             0
```

A total of 10scores were truncated.

### Descriptives

Only variables from the original analysis included. Columns with ".rep" are values from this replication, while columns with ".orig" are values from Palermo.

Code

| subscale | min.rep | max.rep | mean.rep | sd.rep | n | missing | min.orig | max.orig | mea |
|---|---|---|---|---|---|---|---|---|---|
| age | 18 | 37.00 | 22.87 | 3.74 | 156 | 0 | 18.00 | 35.00 | |
| AQ | 3 | 37.00 | 20.04 | 6.40 | 156 | 0 | 4.00 | 34.00 | |
| BES_aff | 18 | 53.00 | 39.81 | 7.24 | 156 | 0 | 18.00 | 54.00 | |
| BES_cog | 24 | 42.00 | 33.93 | 3.70 | 156 | 0 | 28.00 | 45.00 | |
| DASS_anx | 0 | 16.00 | 4.63 | 3.50 | 156 | 0 | 0.00 | 13.00 | |
| DASS_dep | 0 | 17.00 | 4.48 | 4.06 | 156 | 0 | 0.00 | 14.00 | |
| emo_label | 45 | 92.36 | 75.39 | 8.57 | 156 | 0 | 62.19 | 91.67 | |
| emo_match | 25 | 82.64 | 64.06 | 11.26 | 156 | 0 | 47.00 | 89.00 | |
| EQ | 17 | 70.00 | 41.13 | 11.48 | 156 | 0 | 18.00 | 68.00 | |
| MERT | 24 | 74.36 | 53.16 | 9.01 | 156 | 0 | 39.00 | 68.33 | |

Data Processing
Analysis
Robustness Checks
Exploratory analyses of social anxiety

| subscale | min.rep | max.rep | mean.rep | sd.rep | n | missing | min.orig | max.orig | mea |
|---|---|---|---|---|---|---|---|---|---|
| PANAS_n | 10 | 40.00 | 20.79 | 6.50 | 156 | 0 | 10.00 | 31.00 | |
| PANAS_p | 13 | 49.00 | 30.66 | 7.75 | 156 | 0 | 10.00 | 50.00 | |

There were 156 participants: 113 female and 37 male.

## Correlations

Code

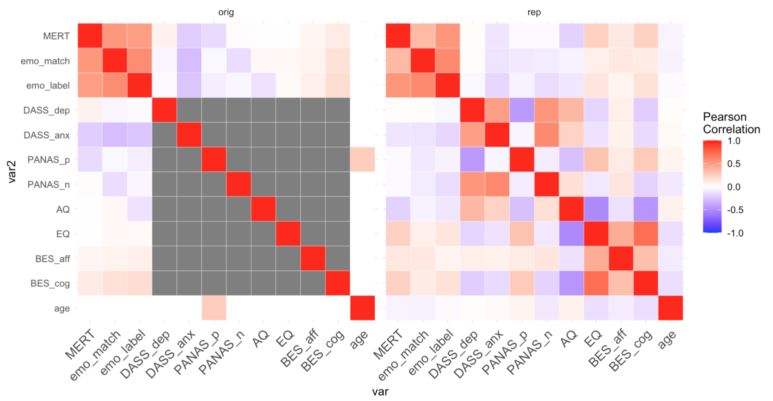


Correlations from the original study and replication (grey cells had no info)

## Cronbach's Alphas

Code

| subscale | alpha |
|---|---|
| AQ | 0.757 |
| BES_cog | 0.619 |
| BES_aff | 0.845 |
| BFNES | 0.918 |
| DASS_dep | 0.869 |
| DASS_s | 0.794 |
| DASS_anx | 0.759 |
| EQ | 0.866 |
| PANAS_p | 0.903 |
| PANAS_n | 0.843 |
| SPS | 0.832 |
| SIAS | 0.808 |

# Analysis

## Hypothesis 1

Code

Scores on the emotion matching task and the DASS anxiety subscale were correlated with r = -0.117; 95% CI = [-0.269, 0.041], p = 0.147.

## Hypothesis 2

Code

Scores on the emotion labeling task and the DASS anxiety subscale were correlated with r = -0.175; 95% CI = [-0.323, -0.018], p = 0.029.

## Hypothesis 3

Code

Data Processing
Analysis
Robustness Checks
Exploratory analyses of social anxiety

| Variable | N | Emotion Matching | | Emotion Labelling | |
|---|---|---|---|---|---|
| | | r | p | r | p |
| AQ | 156 | -0.061 | 0.447 | -0.108 | 0.181 |
| BES_aff | 156 | 0.122 | 0.130 | 0.058 | 0.473 |
| BES_cog | 156 | 0.103 | 0.201 | 0.153 | 0.057 |
| DASS_dep | 156 | 0.018 | 0.826 | -0.035 | 0.665 |
| EQ | 156 | 0.085 | 0.290 | 0.130 | 0.106 |
| PANAS_n | 156 | -0.088 | 0.273 | -0.149 | 0.063 |
| PANAS_p | 156 | -0.094 | 0.242 | -0.055 | 0.494 |

## Hypothesis 4

Code

| Variable | N | MERT | |
|---|---|---|---|
| | | r | p |
| AQ | 156 | -0.195 | 0.015 |
| BES_aff | 156 | 0.115 | 0.153 |
| BES_cog | 156 | 0.237 | 0.003 |
| DASS_anx | 156 | -0.112 | 0.163 |
| DASS_dep | 156 | 0.013 | 0.868 |
| EQ | 156 | 0.238 | 0.003 |
| PANAS_n | 156 | -0.020 | 0.808 |
| PANAS_p | 156 | -0.024 | 0.765 |

# Robustness Checks

## Truncated to Original Range

Truncate data to the original range from Table 1 in Palermo et al (2018).

Code

Code

| subscale | min.rep | max.rep | mean.rep | sd.rep | n | missing | min.orig | max.orig | mea |
|---|---|---|---|---|---|---|---|---|---|
| age | 18.00 | 34.00 | 22.72 | 3.59 | 106 | 0 | 18.00 | 35.00 | |
| AQ | 6.00 | 33.00 | 19.32 | 5.71 | 106 | 0 | 4.00 | 34.00 | |
| BES_aff | 18.00 | 53.00 | 40.14 | 7.34 | 106 | 0 | 18.00 | 54.00 | |
| BES_cog | 28.00 | 41.00 | 34.36 | 3.05 | 106 | 0 | 28.00 | 45.00 | |
| DASS_anx | 0.00 | 13.00 | 3.93 | 2.92 | 106 | 0 | 0.00 | 13.00 | |
| DASS_dep | 0.00 | 13.00 | 3.61 | 3.17 | 106 | 0 | 0.00 | 14.00 | |
| emo_label | 63.89 | 90.28 | 77.59 | 6.15 | 106 | 0 | 62.19 | 91.67 | |
| emo_match | 50.69 | 82.64 | 67.73 | 7.19 | 106 | 0 | 47.00 | 89.00 | |
| EQ | 20.00 | 68.00 | 41.32 | 10.74 | 106 | 0 | 18.00 | 68.00 | |
| MERT | 39.74 | 67.95 | 54.34 | 7.01 | 106 | 0 | 39.00 | 68.33 | |

### Hypothesis 1

Code

For data truncated to the original range from Palermo et al. (2018), scores on the emotion matching task and the DASS anxiety subscale were correlated with r = -0.161; 95% CI = [-0.342, 0.03], p = 0.098.

Data Processing
Analysis
Robustness Checks
Exploratory analyses of social anxiety

### Hypothesis 2

Code

For data truncated to the original range from Palermo et al. (2018), scores on the emotion labeling task and the DASS anxiety subscale were correlated with r = -0.245; 95% CI = [-0.416, -0.057], p = 0.011.

### Hypothesis 3

Code

| Variable | N | Emotion Matching | | Emotion Labelling | |
|---|---|---|---|---|---|
| | | r | p | r | p |
| AQ | 106 | -0.055 | 0.579 | -0.104 | 0.288 |
| BES_aff | 106 | 0.119 | 0.224 | -0.045 | 0.644 |
| BES_cog | 106 | 0.097 | 0.321 | 0.057 | 0.565 |
| DASS_dep | 106 | -0.004 | 0.969 | -0.065 | 0.506 |
| EQ | 106 | 0.195 | 0.045 | 0.154 | 0.114 |
| PANAS_n | 106 | -0.103 | 0.295 | -0.160 | 0.101 |
| PANAS_p | 106 | -0.061 | 0.536 | -0.070 | 0.478 |

### Hypothesis 4

Code

| Variable | N | MERT | |
|---|---|---|---|
| | | r | p |
| AQ | 106 | -0.300 | 0.002 |
| BES_aff | 106 | 0.106 | 0.279 |
| BES_cog | 106 | 0.334 | 0.000 |
| DASS_anx | 106 | -0.148 | 0.131 |
| DASS_dep | 106 | 0.013 | 0.893 |
| EQ | 106 | 0.277 | 0.004 |
| PANAS_n | 106 | -0.027 | 0.781 |
| PANAS_p | 106 | 0.138 | 0.157 |

## Emotion Matching

Code

Scores on the DASS anxiety subscale negatively predicted scores on the emotion matching task (estimate = -0.375; 95% CI = [-0.883, 0.133]; t = -1.457; p = 0.147).

### Controlling for Sex

Code

Controlling for sex, Scores on the DASS anxiety subscale negatively predicted scores on the emotion matching task (estimate = -0.494; 95% CI = [-1.009, 0.021]; t = -1.896; p = 0.060).

### Controlling for Age

Code

Controlling for age, Scores on the DASS anxiety subscale negatively predicted scores on the emotion matching task (estimate = -0.37; 95% CI = [-0.879, 0.14]; t = -1.434; p = 0.154).

### Controlling for Sex and Age

Code

Controlling for sex, age and their interaction, Scores on the DASS anxiety subscale negatively predicted scores on the emotion matching task (estimate = -0.467; 95% CI = [-0.984, 0.05]; t = -1.786; p = 0.076).

## Emotion Labeling

Code

Scores on the DASS anxiety subscale negatively predicted scores on the emotion labelling

Data Processing
Analysis
Robustness Checks
Exploratory analyses of social anxiety

task (estimate = -0.429; 95% CI = [-0.812, -0.045]; t = -2.206; p = 0.029).

### Controlling for Sex

Code

Controlling for sex, Scores on the DASS anxiety subscale negatively predicted scores on the emotion labelling task (estimate = -0.51; 95% CI = [-0.898, -0.122]; t = -2.6; p = 0.010).

### Controlling for Age

Code

Controlling for age, Scores on the DASS anxiety subscale negatively predicted scores on the emotion labelling task (estimate = -0.427; 95% CI = [-0.812, -0.042]; t = -2.19; p = 0.030).

### Controlling for Sex and Age

Code

Controlling for sex, age and their interaction, Scores on the DASS anxiety subscale negatively predicted scores on the emotion labelling task (estimate = -0.503; 95% CI = [-0.894, -0.112]; t = -2.54; p = 0.012).

## MERT

Code

Scores on the DASS anxiety subscale negatively predicted scores on the MERT (estimate = -0.289; 95% CI = [-0.696, 0.119]; t = -1.401; p = 0.163).

### Controlling for Sex

Code

Controlling for sex, Scores on the DASS anxiety subscale negatively predicted scores on the MERT (estimate = -0.324; 95% CI = [-0.735, 0.087]; t = -1.557; p = 0.122).

### Controlling for Age

Code

Controlling for age, Scores on the DASS anxiety subscale negatively predicted scores on the MERT (estimate = -0.285; 95% CI = [-0.693, 0.124]; t = -1.378; p = 0.170).

### Controlling for Sex and Age

Code

Controlling for sex, age and their interaction, Scores on the DASS anxiety subscale negatively predicted scores on the MERT (estimate = -0.32; 95% CI = [-0.734, 0.095]; t = -1.526; p = 0.129).

## PCA

Palermo et al. “combined the scores across the three tasks using principal components analysis (PCA) to derive a measure of general ‘emotion recognition’, independent of the task”.

Code

| **rowname** | **PC1** |
|---|---|
| emo_match | 0.80 |
| emo_label | 0.88 |
| MERT | 0.76 |

The proportion of variance explained by this principal component was 66.7%.

Code

| **Variable** | **N** | **PC** | |
|---|---|---|---|
| | | **r** | **p** |
| AQ | 156 | -0.146 | 0.069 |
| BES_aff | 156 | 0.118 | 0.143 |
| BES_cog | 156 | 0.199 | 0.013 |
| DASS_anx | 156 | -0.167 | 0.038 |
| DASS_dep | 156 | -0.003 | 0.968 |
| EQ | 156 | 0.182 | 0.023 |
| PANAS_n | 156 | -0.109 | 0.177 |

Data Processing
Analysis
Robustness Checks
Exploratory analyses of social anxiety

| Variable | N | PC | |
|---|---|---|---|
| | | r | p |
| PANAS_p | 156 | -0.071 | 0.377 |

# Exploratory analyses of social anxiety

Although data on social anxiety specifically were not collected by Palermo et al. (2018), some researchers have suggested that because of fears concerning negative evaluation, social anxiety may be a key correlate of individual differences in emotion recognition (e.g., Rapee & Heimberg, 1997; Hirsch & Clark, 2004). Consequently, we will repeat the analyses described in Hypotheses 1, 2, and 4 (and the related robustness checks) using scores on the Brief Fear of Negative Evaluation Scale (BFNE; Leary, 1983) and the 6-item versions of the Social Interaction Anxiety Scale (SIAS) and Social Phobia Scale (SPS) developed by Lorna et al. (2012).

## Hypotheses 1, 2, 4

Code

| Variable | N | Emotion Matching | | Emotion Labelling | | MERT | |
|---|---|---|---|---|---|---|---|
| | | r | p | r | p | r | p |
| BFNES | 154 | 0.165 | 0.041 | 0.021 | 0.792 | 0.099 | 0.220 |
| SIAS | 156 | 0.058 | 0.469 | 0.010 | 0.899 | 0.031 | 0.696 |
| SPS | 155 | -0.017 | 0.838 | -0.064 | 0.430 | -0.066 | 0.417 |

## Truncated to Original Paper Range

Code

| Variable | N | Emotion Matching | | Emotion Labelling | | MERT | |
|---|---|---|---|---|---|---|---|
| | | r | p | r | p | r | p |
| BFNES | 126 | 0.050 | 0.580 | -0.014 | 0.875 | 0.054 | 0.549 |
| SIAS | 128 | -0.053 | 0.555 | -0.014 | 0.879 | 0.006 | 0.943 |
| SPS | 127 | -0.112 | 0.210 | -0.076 | 0.395 | -0.124 | 0.165 |

## Linear Models

Controlling for sex, age, and sex*age

Code

### Emotion Matching

Code

| Variable | control | n | estimate | se | lower CI | upper CI | t | p |
|---|---|---|---|---|---|---|---|---|
| BFNES | | 154 | 0.159 | 0.083 | -0.004 | 0.322 | 1.922 | 0.056 |
| BFNES | age_c | 154 | 0.157 | 0.083 | -0.006 | 0.321 | 1.900 | 0.059 |
| BFNES | sex_e | 154 | 0.148 | 0.084 | -0.018 | 0.313 | 1.762 | 0.080 |
| BFNES | sex_e*age_c | 154 | 0.151 | 0.084 | -0.015 | 0.317 | 1.802 | 0.074 |
| SIAS | | 156 | 0.176 | 0.188 | -0.194 | 0.547 | 0.940 | 0.349 |
| SIAS | age_c | 156 | 0.178 | 0.188 | -0.193 | 0.549 | 0.948 | 0.344 |
| SIAS | sex_e | 156 | 0.144 | 0.190 | -0.232 | 0.520 | 0.757 | 0.450 |
| SIAS | sex_e*age_c | 156 | 0.159 | 0.190 | -0.216 | 0.535 | 0.838 | 0.403 |
| SPS | | 155 | 0.013 | 0.172 | -0.327 | 0.354 | 0.078 | 0.938 |
| SPS | age_c | 155 | 0.021 | 0.173 | -0.321 | 0.363 | 0.123 | 0.902 |
| SPS | sex_e | 155 | -0.026 | 0.173 | -0.368 | 0.316 | -0.152 | 0.880 |
| SPS | sex_e*age_c | 155 | -0.006 | 0.174 | -0.349 | 0.338 | -0.032 | 0.974 |

Data Processing
Analysis
Robustness Checks
Exploratory analyses of social anxiety

### Emotion Labeling

Code

| Variable | control | n | estimate | se | lower CI | upper CI | t | p |
|---|---|---|---|---|---|---|---|---|
| BFNES | | 154 | 0.026 | 0.064 | -0.100 | 0.152 | 0.411 | 0.682 |
| BFNES | age_c | 154 | 0.026 | 0.064 | -0.101 | 0.152 | 0.399 | 0.690 |
| BFNES | sex_e | 154 | 0.003 | 0.064 | -0.124 | 0.130 | 0.050 | 0.961 |
| BFNES | sex_e*age_c | 154 | 0.004 | 0.065 | -0.124 | 0.132 | 0.058 | 0.954 |
| SIAS | | 156 | 0.035 | 0.143 | -0.248 | 0.318 | 0.247 | 0.805 |
| SIAS | age_c | 156 | 0.036 | 0.144 | -0.248 | 0.320 | 0.251 | 0.802 |
| SIAS | sex_e | 156 | -0.003 | 0.145 | -0.289 | 0.284 | -0.018 | 0.986 |
| SIAS | sex_e*age_c | 156 | 0.002 | 0.146 | -0.286 | 0.290 | 0.014 | 0.989 |
| SPS | | 155 | -0.094 | 0.131 | -0.353 | 0.166 | -0.714 | 0.476 |
| SPS | age_c | 155 | -0.091 | 0.132 | -0.352 | 0.170 | -0.689 | 0.492 |
| SPS | sex_e | 155 | -0.110 | 0.132 | -0.371 | 0.150 | -0.836 | 0.404 |
| SPS | sex_e*age_c | 155 | -0.103 | 0.133 | -0.366 | 0.160 | -0.773 | 0.441 |

### MERT

Code

| Variable | control | n | estimate | se | lower CI | upper CI | t | p |
|---|---|---|---|---|---|---|---|---|
| BFNES | | 154 | 0.097 | 0.067 | -0.035 | 0.228 | 1.452 | 0.149 |
| BFNES | age_c | 154 | 0.096 | 0.067 | -0.037 | 0.228 | 1.429 | 0.155 |
| BFNES | sex_e | 154 | 0.075 | 0.067 | -0.057 | 0.208 | 1.124 | 0.263 |
| BFNES | sex_e*age_c | 154 | 0.074 | 0.068 | -0.060 | 0.207 | 1.095 | 0.275 |
| SIAS | | 156 | 0.098 | 0.150 | -0.200 | 0.395 | 0.649 | 0.517 |
| SIAS | age_c | 156 | 0.099 | 0.151 | -0.199 | 0.397 | 0.657 | 0.512 |
| SIAS | sex_e | 156 | 0.042 | 0.151 | -0.257 | 0.341 | 0.276 | 0.783 |
| SIAS | sex_e*age_c | 156 | 0.043 | 0.152 | -0.259 | 0.344 | 0.280 | 0.780 |
| SPS | | 155 | -0.109 | 0.138 | -0.382 | 0.163 | -0.792 | 0.429 |
| SPS | age_c | 155 | -0.103 | 0.139 | -0.376 | 0.171 | -0.741 | 0.460 |
| SPS | sex_e | 155 | -0.120 | 0.137 | -0.391 | 0.152 | -0.870 | 0.386 |
| SPS | sex_e*age_c | 155 | -0.112 | 0.139 | -0.386 | 0.162 | -0.807 | 0.421 |

````
---
title: 'Emotion Recognition RR'
output:
  html_document:
    toc: true
    toc_float: true
    toc_depth: 4
    code_folding: hide
---

```{r setup, include=FALSE}
knitr::opts_chunk$set(echo = TRUE)
library(tidyverse)
library(readxl)
library(pwr)
library(kableExtra)
library(psych) # for SPSS-style PCA
library(faux) # devtools::install_github("debruine/faux")
```

```{r functions}
# round up from .xxx5 instead of rounding to even number
aparound <- function(n, digits = 0) {
  round(n + 1e-10, digits)
}

# function to format p-values
pval <- function(p, digits = 3) {
  if (p < .001) return("p < .001")

  sprintf(paste0("p = %.", digits, "f"), aparound(p, digits))
}

# calculate p-value from r and n
r_to_p <- function(r, n) {
  t <- abs(r) / sqrt((1-r^2)/(n-2))
  2*pt(t, df = n-2, lower = FALSE)
}
```


### Sample Size

```{r power}
smallest_effect <- pwr::pwr.r.test(n = 160,
                                   sig.level = 0.05,
                                   power = 0.8,
                                   alternative = "two.sided")$r %>%
                   round(3)
```

Sample size is set at $n = 160$, following the small telescopes
approach, which sets replication sample size at 2.5 times the
original sample size ($n = 63$). With $alpha = .05$, this design has
````

80% power for two-tailed tests to detect correlations as small as $|
r| = `r smallest_effect`$.

## Data Processing

### Simulate data

Descriptive data are taken from from Palermo et al (2018) with correlation structure from correlations reported in Palermo et al. (2018) or set to r = 0.5 for all depression/anxiety measures (as unreported in Palermo).

```{r}
# descriptive data from Palermo et al 2018
orig_desc <- tibble(
  vars = c("MERT", "emo_match", "emo_label", "DASS_dep", "DASS_anx",
           "PANAS_p", "PANAS_n",  "AQ", "EQ",
           "BES_aff", "BES_cog", "age"),
  min =  c(   39,    47, 62.19,    0,    0,    10,    10,     4,
18,    18,    28, 18),
  max =  c(68.33,    89, 91.67,   14,   13,    50,    31,    34,
68,    54,    45, 35),
  mean = c(54.73, 73.28, 77.83, 4.17, 3.05, 29.13, 17.60, 16.20,
44.56, 40.40, 36.90, 21),
  sd =   c( 6.55,  8.19,  6.04, 3.74, 3.18,  6.06,  4.96,  5.78,
10.61,  7.26,  3.37, 5)
)

n <- 160
# correlations from Palermo et al 2018
# all .5 are guesses (assuming positive correlations among measures
# all 0s are unspecified null effects "(all r < .09)" for age.
orig_cors <-
        c(.53, .50,  .070, -.216, -.159,  .017, -.010, -.004, .
048, .104, 0,  # MERT
               .58, -.041, -.287, -.035, -.147,  .035,  .039, .
064, .157, 0,  # emo_match
                    -.020, -.255, -.071, -.038, -.123,  .032, .
082, .178, 0,  # emo_label
                           .5,    .5,    .5,    .5,    .5,   .
5,   .5,   0,  # DASS_depression
                                  .5,    .5,    .5,    .5,   .
5,   .5,   0,  # DASS_anxiety
                                         .5,    .5,    .5,   .
5,   .5, .26,  # PANAS_p
                                                .5,    .5,   .
5,   .5,   0,  # PANAS_n
                                                       .5,   .
5,   .5,   0,  # AQ
                                                             .
5,   .5,   0,  # EQ

.5,   0,  # BES_aff
```

````
0)  # BES_cog

dat <- rnorm_multi(n, vars = nrow(orig_desc),
                  mu = orig_desc$mean,
                  sd = orig_desc$sd,
                  r = orig_cors,
                  varnames = orig_desc$vars) %>%
       mutate(sex = sample(c("m", "f"), n, replace = T, prob = c(.
45, .55)),
              sex_e = recode(sex, "m" = 0.5, "f" = -0.5),
              age = sample(18:25, nrow(.), replace = T),
              age_c = age - mean(age))

```

### Load Data

```{r}
colnames <- c("Response ID", "Date submitted", "Last page", "Start
language", "Date started", "Date last action", "ID Code",
paste0("example_", 1:13),
              expand.grid(c("listen", "choose", "if"), 1:78) %>%
                unite(V, Var1, Var2) %>% pull(V))

mert_raw <- read_xls("data/results-survey754687 (1).xls", col_names
= colnames, skip = 1)

exp_raw <- read_csv("data/Palermo-Replication-RSOS-
exps_2020-04-21.csv")
quest_raw <- read_csv("data/Palermo-Replication-RSOS-
quests_2020-04-21.csv", col_types = cols(dv = col_character()))


```

#### Process questionnaire data

```{r}

quest_processed <- quest_raw %>%
  distinct(.keep_all = TRUE) %>% # remove any duplicate entries
  mutate(quest_name = case_when( # label questionnaires
    quest_id == 1095 ~ "ID",
    quest_id == 1032 ~ "DASS",
    quest_id == 1033 ~ "PANAS",
    quest_id == 1034 ~ "AQ",
    quest_id == 1036 ~ "BES",
    quest_id == 1037 ~ "EQ",
    quest_id == 1039 ~ "BFNES",
    quest_id == 1040 ~ "SIAS"
  )) %>%
  mutate(question = gsub("(\\s|\\(|\\))", "", q_name) %>%
                   tolower() %>%
                   paste0(quest_name, "_", .)) %>%
````

```
  arrange(session_id, user_id, question, starttime) %>%
  group_by(session_id, question) %>%
  slice(1) %>%
  ungroup() %>%
  group_by(session_id, user_id) %>%
  mutate(dt = min(starttime)) %>%
  ungroup() %>%
  select(-q_name, -quest_name, -quest_id, -q_id, -order,
         -project_id,-starttime, -endtime) %>%
  spread(question, dv, convert = TRUE)
```

#### Filter data

Remove test runs and fix IDs

```{r}
quest_filtered <- quest_processed %>%
  filter(user_status %in% c("guest", "registered"),
         ID_idcode != "test3",
         !is.na(AQ_1),
         ID_idcode != "2435858") %>%
  mutate(
    ID_idcode = toupper(ID_idcode),
    ID_idcode = gsub("O", "0", ID_idcode), # replace letter O with number 0
    ID_idcode = case_when(
      ID_idcode == "ER095,FEMALE,21" ~ "ER095",
      ID_idcode == "ER120,FEMALE,24" ~ "ER120",
      ID_idcode == "ER122 MALE 23" ~ "ER122",
      ID_idcode == "ER15G" ~ "ER159",
      ID_idcode == "ER110" & dt == "2020-02-14 10:04:41" ~ "ER113",
      ID_idcode == "ER015" & dt == "2019-12-17 14:34:35" ~ "ER014",
      ID_idcode == "ER035" & dt == "2020-01-16 12:36:49" ~ "ER038",
      ID_idcode == "ER065" & dt == "2020-01-22 14:04:29" ~ "ER067",
      TRUE ~ ID_idcode
    )
  ) %>%
  select(-session_id, -user_status)

# explore bad IDs
expected_ids <- faux::make_id(169, "ER", 3)
setdiff(quest_filtered$ID_idcode, expected_ids)
setdiff(expected_ids, quest_filtered$ID_idcode)
count(quest_filtered,ID_idcode) %>% filter(n > 1)

```

#### Calculate scores

Recode AQ and EQ

0 = definitely agree
1 = slightly agree

2 = slightly disagree
3 = definitely disagree

AQ: “Definitely agree” or “slightly agree” responses scored 1 point, on the following items: 1, 2, 4, 5, 6, 7, 9, 12, 13, 16, 18, 19, 20, 21, 22, 23, 26, 33, 35, 39, 41, 42, 43, 45, 46.
“Definitely disagree” or “slightly disagree” responses scored 1 point, on the following items: 3, 8, 10, 11, 14, 15, 17, 24, 25, 27, 28, 29, 30, 31, 32, 34, 36, 37, 38, 40, 44, 47, 48, 49, 50.

EQ: “Definitely agree” responses scored 2 points and “slightly agree” responses scored 1 point on the following items: 1, 6, 19, 22, 25, 26, 35, 36, 37, 38, 41, 42, 43, 44, 52, 54, 55, 57, 58, 59, 60.
“Definitely disagree” responses scored 2 points and “slightly disagree” responses scored 1 point on the following items: 4, 8, 10, 11, 12, 14, 15, 18, 21, 27, 28, 29, 32, 34, 39, 46, 48, 49, 50.

````
```{r}
# "0" = 1, "1" = 1, "2" = 0, "3" = 0
aq_agree <- c(1, 2, 4, 5, 6, 7, 9, 12, 13, 16, 18, 19, 20, 21,
              22, 23, 26, 33, 35, 39, 41, 42, 43, 45, 46)
# "0" = 0, "1" = 0, "2" = 1, "3" = 1
aq_disagree <- c(3, 8, 10, 11, 14, 15, 17, 24, 25, 27, 28, 29,
                 30, 31, 32, 34, 36, 37, 38, 40, 44, 47, 48, 49, 50)
# "1" = 2, "2" = 1, "3" = 0, "4" = 0
eq_agree <- c(1, 6, 19, 22, 25, 26, 35, 36, 37, 38, 41, 42, 43,
              44, 52, 54, 55, 57, 58, 59, 60)
# "1" = 0, "2" = 0, "3" = 1, "4" = 2
eq_disagree <- c(4, 8, 10, 11, 12, 14, 15, 18, 21, 27, 28, 29,
                 32, 34, 39, 46, 48, 49, 50)
# reverse code some bfnes
bfnes_rev <- c(2, 4, 7, 10)
# reverse code some BES questions
bes_rev <- c(1, 6, 7, 8, 13, 18, 20)

quest_recoded <- quest_filtered %>%
  mutate(ID = ID_idcode) %>%
  select(-ID_idcode) %>%
  gather(question, value, AQ_1:SIAS_9sps) %>%
  separate(question, c("questionnaire", "question")) %>%
  mutate(subscale = gsub("(\\d|_)", "", question)) %>%
  unite(subscale, questionnaire, subscale) %>%
  mutate(subscale = sub("affectiveempathy", "aff", subscale),
         subscale = sub("cognitiveempathy", "cog", subscale),
         subscale = sub("DASS_a", "DASS_anx", subscale),
         subscale = sub("DASS_d", "DASS_dep", subscale),
         subscale = sub("SIAS_sias", "SIAS", subscale),
         subscale = sub("SIAS_sps", "SPS", subscale),
         subscale = sub("_$", "", subscale)
  ) %>%
  mutate(value = ifelse(subscale == "AQ" & (question %in% aq_agree),
                        recode(value, "0" = 1, "1" = 1, "2" = 0, "3"
````
````

````
= 0,
                              .default = NA_real_), value),
         value = ifelse(subscale == "AQ" & (question %in%
aq_disagree),
                       recode(value, "0" = 0, "1" = 0, "2" = 1, "3"
= 1,
                              .default = NA_real_), value),
         value = ifelse(subscale == "EQ" & (question %in% eq_agree),
                       recode(value, "1" = 2, "2" = 1, "3" = 0, "4"
= 0,
                              .default = NA_real_), value),
         value = ifelse(subscale == "EQ" & (question %in%
eq_disagree),
                       recode(value, "1" = 0, "2" = 0, "3" = 1, "4"
= 2,
                              .default = NA_real_), value),
         value = ifelse(subscale == "BFNES" & (question %in%
bfnes_rev),
                       recode(value, "1" = 5, "2" = 4, "3" = 3, "4"
= 2, "5" = 1,
                              .default = NA_real_), value),
         value = ifelse(subscale %in% c("BES_cog", "BES_aff") &
                         (gsub('\\D+', '', question) %in% bes_rev),
                       recode(value, "1" = 5, "2" = 4, "3" = 3, "4"
= 2, "5" = 1,
                              .default = NA_real_), value)
         )

# generate summary scores
quest_scores <- quest_recoded %>%
  group_by(ID, user_id, user_sex, user_age, subscale) %>%
  summarise(score = sum(value)) %>%
  ungroup()

```



```{r, out.width="100%", fig.width = 10, fig.height = 6}
ggplot(quest_scores, aes(score, fill = subscale)) +
  geom_histogram(binwidth = 1, color = "grey", show.legend = FALSE)
+
  facet_wrap(~subscale, scales = "free")

ggsave("quest_distributions.png", width = 10, height = 6)
```

#### Process Experiment Data

```{r}
exp_processed <- exp_raw %>%
  select(user_id, exp_id, trial_n, trial_name, dv, rt) %>%
  mutate(exp_name = case_when( # label exp
    exp_id == 190 ~ "emo_match_practice",
    exp_id == 188 ~ "emo_match",
````

````
    exp_id == 191 ~ "emo_label_practice",
    exp_id == 189 ~ "emo_label"
  )) %>%
  filter(exp_id %in% c(188, 189)) %>% #remove practice trials
  mutate(trial_name = tolower(trial_name) %>% sub("\\.jpg", "", .))
```

```{r}
matching_key <- readxl::read_xlsx("data/Matching task answer
key.xlsx", sheet = 2, skip = 2) %>%
  mutate(trial_name = sub("\\.jpg", "", `Stimulus name`),
         trial_name = sub("\\&", "_", trial_name) %>% tolower()) %>%
  select(trial_name, answer = `Correct answer`)

expected <- matching_key$trial_name
obs <- exp_processed %>%
  filter(exp_name == "emo_match") %>%
  distinct(trial_name) %>%
  pull(trial_name)

list(
  "expected" = setdiff(expected, obs),
  "observed" = setdiff(obs, expected)
)
```

```{r}
# fix typos
exp_processed <- exp_processed %>%
  mutate(trial_name = case_when(
    trial_name == "026sad_fea09" ~ "026sad_fea01",
    trial_name == "123sur_fear11" ~ "123sur_fea11",
    TRUE ~ trial_name
  ))

matching_key <- matching_key %>%
  mutate(trial_name = case_when(
    trial_name == "076ang_sid04" ~ "076ang_dis04",
    TRUE ~ trial_name
  ))
```


```{r}

labelling_key <- readxl::read_xlsx("data/labelling_macro.xlsm",
n_max = 148) %>%
  separate(Name, c("discard", "trial_name"),sep = ", ") %>%
  select(trial_name, answer = Correct) %>%
  mutate_all(tolower) %>%
  mutate(trial_name = sub("\\.jpg\\)", "", trial_name))

expected <- labelling_key$trial_name
obs <- exp_processed %>%
````

```
  filter(exp_name == "emo_label") %>%
  distinct(trial_name) %>%
  pull(trial_name)

list(
  "expected" = setdiff(expected, obs),
  "observed" = setdiff(obs, expected)
)

```

```{r}
# fix typos
exp_processed <- exp_processed %>%
  mutate(trial_name = case_when(
    trial_name == "023bm06anhl" ~ "023bm29anhl",
    trial_name == "044af22sahl" ~ "044bm11sahl",
    trial_name == "074af07afs" ~ "074bf09afs",
    TRUE ~ trial_name
  ))
```

#### Calculate scores

```{r}

keys <- matching_key %>%
  mutate(answer = as.character(answer)) %>%
  bind_rows(labelling_key)

exp_scores <- exp_processed %>%
  mutate(dv = case_when( # make same as answer key
    dv == "angry" ~ "anger",
    dv == "disgusted" ~ "disgust",
    dv == "fearful" ~ "fear",
    dv == "surprised" ~ "surprise",
    TRUE ~ dv
  )) %>%
  filter(user_id %in% quest_scores$user_id) %>%
  left_join(keys, by = "trial_name") %>%
  mutate(correct = answer == dv) %>% # score correct trials
  mutate(correct = ifelse(is.na(correct), FALSE, correct)) %>% #set
timed-out trials to incorrect
  filter(trial_n > 4) %>% # remove "practice trials"
  group_by(user_id, exp_name) %>%
  summarise(score = mean(correct, na.rm = TRUE) * 100) %>%
  spread(exp_name, score)
```

#### Process MERT data

```{r, message=FALSE}
```

```
mert_raw <- read_xls("data/results-survey754687 (1).xls")

mert_processed <- mert_raw %>%
  select(-(8:20)) %>% # remove training trials
  select(id = `Response ID`,
         dt = `Date started`,
         ID_idcode = `ID Code`,
         tidyselect::starts_with("Please "),
         tidyselect::starts_with("{if")) %>%
  mutate(ID_idcode = trimws(ID_idcode))

colnames <- names(mert_processed)
newnames <- sub("Please choose the term which best describes the
emotion expressed in the preceding ", "", colnames, fixed = TRUE)
%>%
  sub(" clip", "", ., fixed = TRUE) %>%
  sub("\\.+\\d+", "", .) %>%
  sub("{if(", "", ., fixed = TRUE) %>%
  sub('== \"', "_", ., fixed = TRUE) %>%
  sub('\", 1, 0)}', "", ., fixed = TRUE) %>%
  gsub("\\s", "", .)

names(mert_processed) <- paste0(c("", "", "", 1:78, 1:78), "_",
newnames)

```


```{r}
mert1 <- mert_processed %>%
  gather(trial_name, value, `1_audio`:`78_A11205V_A`) %>%
  separate(trial_name, c("trial", "type", "answer"), fill = "right")

mert_values <- filter(mert1, type %in% c("audio", "video" ,
"photo")) %>% select(-answer)
mert_answers <- filter(mert1, !type %in% c("audio", "video" ,
"photo")) %>% select(-type, -value)

mert_joined <- left_join(mert_values, mert_answers,
                  by = c("_id", "_dt", "_ID_idcode", "trial"))
```



```{r, results='asis'}
# figure out what the right answers are
count(mert_joined, value, answer) %>%
  spread(answer, n) %>%
  filter(!is.na(value)) %>%
  kable() %>%
  kable_styling(bootstrap_options = c("striped"))
```

#### Recode MERT

"A" = "Anxiety",

"K" = "Cold anger",
"V" = "Contempt",
"Z" = "Despair",
"E" = "Disgust",
"U" = "Elated joy",
"F" = "Happiness",
"H" = "Hot anger",
"P" = "Panic fear",
"T" = "Sadness"

```{r}

mert <- mert_joined %>%
  mutate(answer = recode(answer, "A" = "Anxiety",
                         "K" = "Cold anger",
                         "V" = "Contempt",
                         "Z" = "Despair",
                         "E" = "Disgust",
                         "U" = "Elated joy",
                         "F" = "Happiness",
                         "H" = "Hot anger",
                         "P" = "Panic fear",
                         "T" = "Sadness")) %>%
  mutate(correct = value == answer) %>%
  select(ID = `_ID_idcode`, dt = `_dt`, correct) %>%
  group_by(ID) %>%
  summarise(MERT = mean(correct, na.rm = TRUE) * 100) %>%
  ungroup() %>%
  mutate(ID = toupper(ID) %>% gsub("O", "0", .)) %>%
  arrange(ID)

# ER143 is missing one trial

```

#### Combine Data

```{r}
combo_dat <- quest_scores %>%
  spread(subscale, score) %>%
  left_join(exp_scores, by = "user_id") %>%
  left_join(mert, by = "ID") %>%
  rename(sex = user_sex, age = user_age) %>%
  mutate(sex_e = recode(sex, "male" = 0.5, "female" = -0.5, .default
= 0),
         age_c = age - mean(age))
```

#### Exclusions

Outliers (scores on a measure that are more than 3 standard deviations from the mean score for that measure) will be adjusted to a score one point higher than the closest non-outlier score (following Palermo et al, 2018). As a positive control, participants

scoring lower than chance on either of the emotion recognition tasks will be excluded from all analyses.

```{r}
# remove participants who score lower than chance or have missing
scores
# matching chance = 1/3
# labelling chance = 1/6
excl_emo <- combo_dat %>%
  filter(emo_match < 1/3 | emo_label < 1/6 | is.na(MERT))

excl_quest <- combo_dat %>%

filter(is.na(AQ+BES_aff+BES_cog+DASS_anx+DASS_dep+EQ+PANAS_n+PANAS_p
))

# exclusions remove 2 who did not complete the matching study
# 1 who did not complete the MERT,
# and 9 with missing Palermo questionnaire data
excl_dat <- combo_dat %>%
  filter(!user_id %in% excl_emo$user_id) %>%
  filter(!user_id %in% excl_quest$user_id)
```

`r nrow(excl_emo)` participants were removed who did not complete an emotion task and `r nrow(excl_quest)` participants who had missing questionnaire data.

```{r}
descr <- excl_dat %>%
  gather(subscale, score, AQ:MERT) %>%
  group_by(subscale) %>%
  summarise(m = mean(score, na.rm = TRUE),
            sd = sd(score, na.rm = TRUE)) %>%
  ungroup() %>%
  mutate(min = m - 3*sd, max = m + 3*sd)

trunc_dat <- excl_dat %>%
  gather(subscale, score, AQ:MERT) %>%
  left_join(descr, by = "subscale") %>%
  mutate(new_score = ifelse(score < min, ceiling(min)-1,
                     ifelse(score > max, floor(max) + 1,
                            score)),
         changed = new_score != score) %>%
  select(-score) %>%
  rename(score = new_score)

n_truncated <- trunc_dat %>%
  group_by(subscale) %>%
  summarise(changed = sum(changed, na.rm = TRUE)) %>%
  print()
```

A total of `r sum(n_truncated$changed)`scores were truncated.

#### Descriptives

Only variables from the original analysis included. Columns with ".rep" are values from this replication, while columns with ".orig" are values from Palermo.

```{r, results='asis', warning=FALSE}
all_dat <- trunc_dat %>%
  select(-changed, -m, -sd, -min, -max) %>%
  spread( subscale, score)

dat <- select(all_dat, -user_id, -ID, -BFNES, - DASS_s, -SIAS, -SPS)

dat %>%
  select(-sex, -sex_e, -age_c) %>%
  gather(subscale, score, age:PANAS_p) %>%
  group_by(subscale) %>%
  summarise(min = min(score, na.rm = TRUE),
            max = max(score, na.rm = TRUE),
            mean = mean(score, na.rm = TRUE),
            sd = sd(score,na.rm = TRUE),
            n = n(),
            missing = sum(is.na(score))) %>%
  left_join(orig_desc, by = c("subscale" = "vars"),
            suffix = c(".rep", ".orig")) %>%
  kable(digits = 2) %>%
  kable_styling(bootstrap_options = c("striped"))
```

There were `r nrow(dat)` participants: `r sum(dat$sex=="female", na.rm = TRUE)` female and `r sum(dat$sex=="male", na.rm = TRUE)` male.

#### Correlations

```{r, fig.cap="Correlations from the original study and replication (grey cells had no info)", fig.width=10, fig.height = 6}
rep_cors <- dat %>%
  select(-sex, -sex_e, -age_c) %>%
  cor(use = "complete.obs") %>%
  as_tibble(rownames = "var") %>%
  mutate_if(is.numeric, aparound, 3)

v <- c("MERT", "emo_match", "emo_label", "DASS_dep", "DASS_anx",
"PANAS_p", "PANAS_n", "AQ", "EQ", "BES_aff", "BES_cog", "age")

faux::cormat_from_triangle(orig_cors) %>%
  as.data.frame() %>%
  set_names(v) %>%
  mutate(var = v) %>%
  left_join(rep_cors, by = "var", suffix = c(".orig", ".rep")) %>%
  gather(var2, score, MERT.orig:age.orig, age.rep:PANAS_p.rep) %>%
  separate(var2, c("var2", "rep"), sep = "\\.") %>%
```

````
  mutate(var = factor(var, levels = v),
         var2 = factor(var2, levels = rev(v)),
         score = ifelse(score == .5 & var != "MERT" & var2 !=
"MERT", NA, score)) %>%
  ggplot(aes(var, var2, fill = score)) +
  geom_tile(color = "white") +
  scale_fill_gradient2(low = "blue", high = "red", mid = "white",
   midpoint = 0, limit = c(-1,1), space = "Lab",
   name="Pearson\nCorrelation") +
  theme_minimal()+
  facet_wrap(~rep) +
  theme(axis.text.x = element_text(angle = 45, vjust = 1,
    size = 12, hjust = 1)) +
  coord_fixed()
```

#### Cronbach's Alphas

```{r results='asis'}

data_alpha <- quest_recoded %>%
  filter(user_id %in% all_dat$user_id) %>%
  select(subscale, user_id, question, value) %>%
  group_by(subscale) %>%
  nest() %>%
  mutate(alpha = map(data, function(d) {
      # calculate cronbach's alpha
      subdata <- d %>%
        select(user_id, question, value) %>%
        as_tibble() %>%
        spread(question, value) %>%
        select(-user_id) %>%
        drop_na()

      capture.output(suppressWarnings(a <- psych::alpha(subdata)))
      a$total["std.alpha"] %>% pluck(1) %>% round(3)
  })) %>%
  select(-data) %>%
  unnest(alpha) %>%
  ungroup()

kable(data_alpha) %>%
  kable_styling(bootstrap_options = c("striped"))
```


## Analysis

### Hypothesis 1

```{r H1}
H1 <- cor.test(dat$DASS_anx, dat$emo_match) %>%
  broom::tidy() %>%
  mutate_if(is.numeric, aparound, 3)
````

```

Scores on the emotion matching task and the DASS anxiety subscale were correlated with r = `r H1$estimate`; 95% CI = [`r H1$conf.low`, `r H1$conf.high`], `r pval(H1$p.value)`.

### Hypothesis 2

```{r H2}
H2 <- cor.test(dat$DASS_anx, dat$emo_label) %>%
  broom::tidy() %>%
  mutate_if(is.numeric, aparound, 3)
```

Scores on the emotion labeling task and the DASS anxiety subscale were correlated with r = `r H2$estimate`; 95% CI = [`r H2$conf.low`, `r H2$conf.high`], `r pval(H2$p.value)`.

### Hypothesis 3

```{r H3, results='asis'}

H3 <- dat %>%
  select(-sex) %>%
  cor() %>%
  as_tibble(rownames = "var") %>%
  select(var, emo_match, emo_label) %>%
  filter(!(var %in% c("emo_match", "emo_label", "DASS_anx", "age",
"age_c", "sex_e", "MERT"))) %>%
  mutate(n = nrow(dat),
         p_match = r_to_p(emo_match, n),
         p_label = r_to_p(emo_label, n)) %>%
  select(var, n, emo_match, p_match, emo_label, p_label)

H3 %>% mutate_if(is.numeric, aparound, 3) %>%
  kable(col.names = c("", "", "r", "p", "r", "p")) %>%
  add_header_above(c("Variable", "N", "Emotion Matching" = 2,
"Emotion Labelling" = 2)) %>%
  kable_styling(bootstrap_options = c("striped"))

```

### Hypothesis 4

```{r H4, results='asis'}

H4 <- dat %>%
  select(-sex) %>%
  cor() %>%
  as_tibble(rownames = "var") %>%
  select(var, MERT) %>%
  filter(!(var %in% c("emo_match", "emo_label", "MERT", "age",
"age_c", "sex_e"))) %>%

````
  mutate(n = nrow(dat), p_mert = r_to_p(MERT, n)) %>%
  select(var, n, MERT, p_mert)

H4 %>% mutate_if(is.numeric, aparound, 3) %>%
  kable(col.names = c("", "", "r", "p")) %>%
  add_header_above(c("Variable", "N", "MERT" = 2)) %>%
  kable_styling(bootstrap_options = c("striped"))
```
````

## Robustness Checks

### Truncated to Original Range

Truncate data to the original range from Table 1 in Palermo et al (2018).

````
```{r truncate-dat}
dat_trunc <- dat %>%
  mutate(id = row_number()) %>%
  gather(vars, val, !!!orig_desc$vars) %>%
  left_join(orig_desc, by = "vars") %>%
  filter(val >= min, val <= max) %>%
  select(-min, -max, -mean, -sd) %>%
  spread(vars, val) %>% # spread to generate NAs in missing values
  gather(vars, val, !!!orig_desc$vars) %>% # gather to filter out
ids with NAs
  group_by(id) %>%
  filter(!is.na(sum(val))) %>%
  ungroup() %>%
  spread(vars, val) %>%
  select(-id)
```

```{r, results='asis', warning=FALSE}
dat_trunc %>%
  select(-sex, -sex_e, -age_c) %>%
  gather(subscale, score, age:MERT) %>%
  group_by(subscale) %>%
  summarise(min = min(score, na.rm = TRUE),
            max = max(score, na.rm = TRUE),
            mean = mean(score, na.rm = TRUE),
            sd = sd(score,na.rm = TRUE),
            n = n(),
            missing = sum(is.na(score))) %>%
  left_join(orig_desc, by = c("subscale" = "vars"),
            suffix = c(".rep", ".orig")) %>%
  kable(digits = 2) %>%
  kable_styling(bootstrap_options = c("striped"))
```
````
````

#### Hypothesis 1

```{r H1-trunc}
H1_trunc <- cor.test(dat_trunc$DASS_anx, dat_trunc$emo_match) %>%
  broom::tidy() %>%
  mutate_if(is.numeric, aparound, 3)
```

For data truncated to the original range from Palermo et al. (2018), scores on the emotion matching task and the DASS anxiety subscale were correlated with r = `r H1_trunc$estimate`; 95% CI = [`r H1_trunc$conf.low`, `r H1_trunc$conf.high`], `r pval(H1_trunc$p.value)`.

#### Hypothesis 2

```{r H2_trunc}
H2_trunc <- cor.test(dat_trunc$DASS_anx, dat_trunc$emo_label) %>%
  broom::tidy() %>%
  mutate_if(is.numeric, aparound, 3)
```

For data truncated to the original range from Palermo et al. (2018), scores on the emotion labeling task and the DASS anxiety subscale were correlated with r = `r H2_trunc$estimate`; 95% CI = [`r H2_trunc$conf.low`, `r H2_trunc$conf.high`], `r pval(H2_trunc$p.value)`.

#### Hypothesis 3

```{r H3_trunc, results='asis'}

H3_trunc <- dat_trunc %>%
  select(-sex) %>%
  cor() %>%
  as_tibble(rownames = "var") %>%
  select(var, emo_match, emo_label) %>%
  filter(!(var %in% c("emo_match", "emo_label", "DASS_anx", "age",
"age_c", "sex_e", "MERT"))) %>%
  mutate(n = nrow(dat_trunc),
         p_match = r_to_p(emo_match, n),
         p_label = r_to_p(emo_label, n)) %>%
  select(var, n, emo_match, p_match, emo_label, p_label)

H3_trunc %>% mutate_if(is.numeric, aparound, 3) %>%
  kable(col.names = c("", "", "r", "p", "r", "p")) %>%
  add_header_above(c("Variable", "N", "Emotion Matching" = 2,
"Emotion Labelling" = 2)) %>%
  kable_styling(bootstrap_options = c("striped"))

```

#### Hypothesis 4

````
```{r H4_trunc, results='asis'}

H4_trunc <- dat_trunc %>%
  select(-sex) %>%
  cor() %>%
  as_tibble(rownames = "var") %>%
  select(var, MERT) %>%
  filter(!(var %in% c("emo_match", "emo_label", "MERT", "age",
"age_c", "sex_e"))) %>%
  mutate(n = nrow(dat_trunc), p_mert = r_to_p(MERT, n)) %>%
  select(var, n, MERT, p_mert)

H4_trunc %>% mutate_if(is.numeric, aparound, 3) %>%
  kable(col.names = c("", "", "r", "p")) %>%
  add_header_above(c("Variable", "N", "MERT" = 2)) %>%
  kable_styling(bootstrap_options = c("striped"))
```
````

### Emotion Matching

````
```{r}
rc_ <- lm(emo_match ~ DASS_anx, data = dat)

rc_stats <- rc_ %>%
  broom::tidy() %>%
  bind_cols(confint(rc_) %>% as_tibble()) %>%
  filter(term == "DASS_anx") %>%
  rename(conf.low = `2.5 %`, conf.high = `97.5 %`) %>%
  mutate_if(is.numeric, aparound, 3)
```
````

Scores on the DASS anxiety subscale negatively predicted scores on the emotion matching task
(estimate = `r rc_stats$estimate`;
95% CI = [`r rc_stats$conf.low`, `r rc_stats$conf.high`];
t = `r (rc_stats$statistic)`;
`r pval(rc_stats$p.value)`).

#### Controlling for Sex

````
```{r}


rc_sex <- lm(emo_match ~ DASS_anx + sex_e, data = dat)

rc_sex_stats <- rc_sex %>%
  broom::tidy() %>%
  bind_cols(confint(rc_sex) %>% as_tibble()) %>%
  filter(term == "DASS_anx") %>%
  rename(conf.low = `2.5 %`, conf.high = `97.5 %`) %>%
  mutate_if(is.numeric, aparound, 3)
````
````

```

Controlling for sex, Scores on the DASS anxiety subscale negatively predicted scores on the emotion matching task
(estimate = `r rc_sex_stats$estimate`;
95% CI = [`r rc_sex_stats$conf.low`, `r rc_sex_stats$conf.high`];
t = `r (rc_sex_stats$statistic)`;
`r pval(rc_sex_stats$p.value)`).

#### Controlling for Age

```{r}
rc_age <- lm(emo_match ~ DASS_anx + age_c, data = dat)

rc_age_stats <- rc_age %>%
  broom::tidy() %>%
  bind_cols(confint(rc_age) %>% as_tibble()) %>%
  filter(term == "DASS_anx") %>%
  rename(conf.low = `2.5 %`, conf.high = `97.5 %`) %>%
  mutate_if(is.numeric, aparound, 3)
```

Controlling for age, Scores on the DASS anxiety subscale negatively predicted scores on the emotion matching task
(estimate = `r rc_age_stats$estimate`;
95% CI = [`r rc_age_stats$conf.low`, `r rc_age_stats$conf.high`];
t = `r (rc_age_stats$statistic)`;
`r pval(rc_age_stats$p.value)`).

#### Controlling for Sex and Age

```{r}
rc_sex_age <- lm(emo_match ~ DASS_anx + sex_e*age_c, data = dat)

rc_sex_age_stats <- rc_sex_age %>%
  broom::tidy() %>%
  bind_cols(confint(rc_sex_age) %>% as_tibble()) %>%
  filter(term == "DASS_anx") %>%
  rename(conf.low = `2.5 %`, conf.high = `97.5 %`) %>%
  mutate_if(is.numeric, aparound, 3)

```

Controlling for sex, age and their interaction, Scores on the DASS anxiety subscale negatively predicted scores on the emotion matching task
(estimate = `r rc_sex_age_stats$estimate`;
95% CI = [`r rc_sex_age_stats$conf.low`, `r rc_sex_age_stats$conf.high`];
t = `r (rc_sex_age_stats$statistic)`;
```

`r pval(rc_sex_age_stats$p.value)`).

### Emotion Labeling

```{r}
rc_ <- lm(emo_label ~ DASS_anx, data = dat)

rc_stats <- rc_ %>%
  broom::tidy() %>%
  bind_cols(confint(rc_) %>% as_tibble()) %>%
  filter(term == "DASS_anx") %>%
  rename(conf.low = `2.5 %`, conf.high = `97.5 %`) %>%
  mutate_if(is.numeric, aparound, 3)
```

Scores on the DASS anxiety subscale negatively predicted scores on the emotion labelling task
(estimate = `r rc_stats$estimate`;
95% CI = [`r rc_stats$conf.low`, `r rc_stats$conf.high`];
t = `r (rc_stats$statistic)`;
`r pval(rc_stats$p.value)`).

#### Controlling for Sex

```{r}


rc_sex <- lm(emo_label ~ DASS_anx + sex_e, data = dat)

rc_sex_stats <- rc_sex %>%
  broom::tidy() %>%
  bind_cols(confint(rc_sex) %>% as_tibble()) %>%
  filter(term == "DASS_anx") %>%
  rename(conf.low = `2.5 %`, conf.high = `97.5 %`) %>%
  mutate_if(is.numeric, aparound, 3)
```

Controlling for sex, Scores on the DASS anxiety subscale negatively predicted scores on the emotion labelling task
(estimate = `r rc_sex_stats$estimate`;
95% CI = [`r rc_sex_stats$conf.low`, `r rc_sex_stats$conf.high`];
t = `r (rc_sex_stats$statistic)`;
`r pval(rc_sex_stats$p.value)`).

#### Controlling for Age

```{r}
rc_age <- lm(emo_label ~ DASS_anx + age_c, data = dat)

rc_age_stats <- rc_age %>%
```

```
  broom::tidy() %>%
  bind_cols(confint(rc_age) %>% as_tibble()) %>%
  filter(term == "DASS_anx") %>%
  rename(conf.low = `2.5 %`, conf.high = `97.5 %`) %>%
  mutate_if(is.numeric, aparound, 3)
```

Controlling for age, Scores on the DASS anxiety subscale negatively predicted scores on the emotion labelling task
(estimate = `r rc_age_stats$estimate`;
95% CI = [`r rc_age_stats$conf.low`, `r rc_age_stats$conf.high`];
t = `r (rc_age_stats$statistic)`;
`r pval(rc_age_stats$p.value)`).

#### Controlling for Sex and Age

```{r}
rc_sex_age <- lm(emo_label ~ DASS_anx + sex_e*age_c, data = dat)

rc_sex_age_stats <- rc_sex_age %>%
  broom::tidy() %>%
  bind_cols(confint(rc_sex_age) %>% as_tibble()) %>%
  filter(term == "DASS_anx") %>%
  rename(conf.low = `2.5 %`, conf.high = `97.5 %`) %>%
  mutate_if(is.numeric, aparound, 3)

```

Controlling for sex, age and their interaction, Scores on the DASS anxiety subscale negatively predicted scores on the emotion labelling task
(estimate = `r rc_sex_age_stats$estimate`;
95% CI = [`r rc_sex_age_stats$conf.low`, `r rc_sex_age_stats$conf.high`];
t = `r (rc_sex_age_stats$statistic)`;
`r pval(rc_sex_age_stats$p.value)`).

### MERT

```{r}
rc_ <- lm(MERT ~ DASS_anx, data = dat)

rc_stats <- rc_ %>%
  broom::tidy() %>%
  bind_cols(confint(rc_) %>% as_tibble()) %>%
  filter(term == "DASS_anx") %>%
  rename(conf.low = `2.5 %`, conf.high = `97.5 %`) %>%
  mutate_if(is.numeric, aparound, 3)
```

Scores on the DASS anxiety subscale negatively predicted scores on the MERT

(estimate = `r rc_stats$estimate`;
95% CI = [`r rc_stats$conf.low`, `r rc_stats$conf.high`];
t = `r (rc_stats$statistic)`;
`r pval(rc_stats$p.value)`).

#### Controlling for Sex

```{r}

rc_sex <- lm(MERT ~ DASS_anx + sex_e, data = dat)

rc_sex_stats <- rc_sex %>%
  broom::tidy() %>%
  bind_cols(confint(rc_sex) %>% as_tibble()) %>%
  filter(term == "DASS_anx") %>%
  rename(conf.low = `2.5 %`, conf.high = `97.5 %`) %>%
  mutate_if(is.numeric, aparound, 3)
```


Controlling for sex, Scores on the DASS anxiety subscale negatively
predicted scores on the MERT
(estimate = `r rc_sex_stats$estimate`;
95% CI = [`r rc_sex_stats$conf.low`, `r rc_sex_stats$conf.high`];
t = `r (rc_sex_stats$statistic)`;
`r pval(rc_sex_stats$p.value)`).



#### Controlling for Age

```{r}
rc_age <- lm(MERT ~ DASS_anx + age_c, data = dat)

rc_age_stats <- rc_age %>%
  broom::tidy() %>%
  bind_cols(confint(rc_age) %>% as_tibble()) %>%
  filter(term == "DASS_anx") %>%
  rename(conf.low = `2.5 %`, conf.high = `97.5 %`) %>%
  mutate_if(is.numeric, aparound, 3)
```

Controlling for age, Scores on the DASS anxiety subscale negatively
predicted scores on the MERT
(estimate = `r rc_age_stats$estimate`;
95% CI = [`r rc_age_stats$conf.low`, `r rc_age_stats$conf.high`];
t = `r (rc_age_stats$statistic)`;
`r pval(rc_age_stats$p.value)`).


#### Controlling for Sex and Age

```{r}
rc_sex_age <- lm(MERT ~ DASS_anx + sex_e*age_c, data = dat)
```

````
rc_sex_age_stats <- rc_sex_age %>%
  broom::tidy() %>%
  bind_cols(confint(rc_sex_age) %>% as_tibble()) %>%
  filter(term == "DASS_anx") %>%
  rename(conf.low = `2.5 %`, conf.high = `97.5 %`) %>%
  mutate_if(is.numeric, aparound, 3)

```

Controlling for sex, age and their interaction, Scores on the DASS
anxiety subscale negatively predicted scores on the MERT
(estimate = `r rc_sex_age_stats$estimate`;
95% CI = [`r rc_sex_age_stats$conf.low`, `r
rc_sex_age_stats$conf.high`];
t = `r (rc_sex_age_stats$statistic)`;
`r pval(rc_sex_age_stats$p.value)`).


### PCA

Palermo et al. "combined the scores across the three tasks using
principal components analysis (PCA) to derive a measure of general
‘emotion recognition’, independent of the task".

```{r, results='asis'}

emo_tests <- select(dat, emo_match, emo_label, MERT)

# principal components analysis (SPSS-style)
ev <- eigen(cor(emo_tests))$values
nfactors <- sum(ev > 1)

pca <- principal(
  emo_tests,
  nfactors=nfactors,
  rotate="none"
)

pvar <- pca$Vaccounted["Proportion Var", ] %>% round(3) * 100

unclass(pca$loadings) %>%
  as.data.frame() %>%
  rownames_to_column() %>%
  kable(digits = 2) %>%
  kable_styling(bootstrap_options = c("striped"))

```


The proportion of variance explained by this principal component was
`r pvar`%.

```{r PC-cors, results='asis'}
````

```
PC_cors <- dat %>%
  mutate(PC = pca$scores) %>%
  select(-sex) %>%
  cor() %>%
  as_tibble(rownames = "var") %>%
  select(var, PC) %>%
  filter(!(var %in% c("emo_match", "emo_label", "MERT", "age",
"age_c", "sex_e", "PC"))) %>%
  mutate(n = nrow(dat),
         p = r_to_p(PC, n)) %>%
  select(var, n, PC, p)

PC_cors %>% mutate_if(is.numeric, aparound, 3) %>%
  kable(col.names = c("", "", "r", "p")) %>%
  add_header_above(c("Variable", "N", "PC" = 2)) %>%
  kable_styling(bootstrap_options = c("striped"))
```

## Exploratory analyses of social anxiety

Although data on social anxiety specifically were not collected by Palermo et al. (2018), some researchers have suggested that because of fears concerning negative evaluation, social anxiety may be a key correlate of individual differences in emotion recognition (e.g., Rapee & Heimberg, 1997; Hirsch & Clark, 2004). Consequently, we will repeat the analyses described in Hypotheses 1, 2, and 4 (and the related robustness checks) using scores on the Brief Fear of Negative Evaluation Scale (BFNE; Leary, 1983) and the 6-item versions of the Social Interaction Anxiety Scale (SIAS) and Social Phobia Scale (SPS) developed by Lorna et al. (2012).

### Hypotheses 1, 2, 4

```{r supplemental, results='asis'}

# select new variables
dat_b <- select(all_dat, sex, age, age_c, sex_e, emo_match,
emo_label, MERT, BFNES, SIAS, SPS)

# some missing values, so calculate n for each variable
dat_n <- dat_b %>%
  select(BFNES, SIAS, SPS) %>%
  gather(var, val, BFNES:SPS) %>%
  group_by(var) %>%
  summarise(n = sum(!is.na(val)))

sup1 <- dat_b %>%
  select(-sex) %>%
  cor(use = "complete.obs") %>%
  as_tibble(rownames = "var") %>%
  select(var, emo_match, emo_label, MERT) %>%
  filter(!(var %in% c("emo_match", "emo_label", "age", "age_c",
```

````
"sex_e", "MERT"))) %>%
  left_join(dat_n, by = "var") %>%
  mutate(p_match = r_to_p(emo_match, n),
         p_label = r_to_p(emo_label, n),
         p_mert = r_to_p(MERT, n)) %>%
  select(var, n, emo_match, p_match, emo_label, p_label, MERT,
p_mert)

sup1 %>% mutate_if(is.numeric, aparound, 3) %>%
  kable(col.names = c("", "", "r", "p", "r", "p", "r","p")) %>%
  add_header_above(c("Variable", "N", "Emotion Matching" = 2,
"Emotion Labelling" = 2, "MERT" = 2)) %>%
  kable_styling(bootstrap_options = c("striped"))

```

### Truncated to Original Paper Range

```{r sup_trunc, results='asis'}

# select new variables
trunc_dat_b <- select(all_dat, sex, age, age_c, sex_e, emo_match,
emo_label, MERT, BFNES, SIAS, SPS) %>%
  filter(emo_match > filter(orig_desc, vars == "emo_match")$min,
         emo_match < filter(orig_desc, vars == "emo_match")$max,
         emo_label > filter(orig_desc, vars == "emo_label")$min,
         emo_label < filter(orig_desc, vars == "emo_label")$max,
         MERT > filter(orig_desc, vars == "MERT")$min,
         MERT < filter(orig_desc, vars == "MERT")$max)


# some missing values, so calculate n for each variable
trunc_dat_n <- trunc_dat_b %>%
  select(BFNES, SIAS, SPS) %>%
  gather(var, val, BFNES:SPS) %>%
  group_by(var) %>%
  summarise(n = sum(!is.na(val)))

sup_trunc <- trunc_dat_b %>%
  select(-sex) %>%
  cor(use = "complete.obs") %>%
  as_tibble(rownames = "var") %>%
  select(var, emo_match, emo_label, MERT) %>%
  filter(!(var %in% c("emo_match", "emo_label", "age", "age_c",
"sex_e", "MERT"))) %>%
  left_join(trunc_dat_n, by = "var") %>%
  mutate(p_match = r_to_p(emo_match, n),
         p_label = r_to_p(emo_label, n),
         p_mert = r_to_p(MERT, n)) %>%
  select(var, n, emo_match, p_match, emo_label, p_label, MERT,
p_mert)

sup_trunc %>% mutate_if(is.numeric, aparound, 3) %>%
  kable(col.names = c("", "", "r", "p", "r", "p", "r","p")) %>%
````

```
  add_header_above(c("Variable", "N", "Emotion Matching" = 2,
"Emotion Labelling" = 2, "MERT" = 2)) %>%
  kable_styling(bootstrap_options = c("striped"))

```

### Linear Models

Controlling for sex, age, and sex*age

```{r}
tasks <- c("emo_match", "emo_label", "MERT")
vars <- c("BFNES", "SIAS", "SPS")
controls <- c("", "+ sex_e", "+ age_c", "+ sex_e*age_c")

lm_list <- crossing(task = tasks, var = vars, control = controls)
%>%
  mutate(formula = paste0(task, " ~ ", var, " ", control)) %>%
  left_join(dat_n, by = "var") %>%
  mutate(analysis = map(formula, function(x) {
    a <- lm(as.formula(x), data = dat_b)

    broom::tidy(a) %>%
     bind_cols(confint(a) %>% as_tibble())
  }))%>%
  unnest(analysis) %>%
  filter(term %in% vars) %>%
  select(task, Variable = var, control, n,
         estimate, se = std.error,
         `lower CI` = `2.5 %`,
         `upper CI` = `97.5 %`,
         t = statistic, p = p.value) %>%
  mutate(control = gsub("\\+ ", "", control))
```

#### Emotion Matching

```{r, results='asis'}

lm_list %>%
  filter(task == "emo_match") %>%
  select(-task) %>%
  kable(digits = 3) %>%
  kable_styling(bootstrap_options = c("striped"))

```

#### Emotion Labeling

```{r, results='asis'}

lm_list %>%
```

````
  filter(task == "emo_label") %>%
  select(-task) %>%
  kable(digits = 3) %>%
  kable_styling(bootstrap_options = c("striped"))

```


#### MERT

```{r, results='asis'}

lm_list %>%
  filter(task == "MERT") %>%
  select(-task) %>%
  kable(digits = 3) %>%
  kable_styling(bootstrap_options = c("striped"))

```
````
````
